# Acute depletion of diacylglycerol from the *cis*-Golgi affects localized nuclear envelope morphology during mitosis[S]

**DOI:** 10.1194/jlr.M083899

**Published:** 2018-06-12

**Authors:** Gary Hong Chun Chung, Marie-Charlotte Domart, Christopher Peddie, Judith Mantell, Kieran Mclaverty, Angela Arabiotorre, Lorna Hodgson, Richard D. Byrne, Paul Verkade, Kenton Arkill, Lucy M. Collinson, Banafshé Larijani

**Affiliations:** Cell Biophysics Laboratory,* Ikerbasque Basque Foundation for Science, Research Centre for Experimental Marine Biology and Biotechnology (PiE) and Biofísika Institute (UPV/EHU, CSIC), University of the Basque Country, Barrio Sarriena s/n 48940, Leioa, Spain; Electron Microscopy Science Technology Platform,† Francis Crick Institute, London, United Kingdom; School of Biochemistry, Faculty of Biomedical Sciences,§ University of Bristol, Bristol, United Kingdom; Wolfson Bioimaging Facility,** University of Bristol, Bristol, United Kingdom; Division of Cancer and Stem Cells,†† The Queen’s Medical Centre, University of Nottingham, Nottingham, United Kingdom

**Keywords:** correlative light electron microscopy, serial block face scanning electron microscopy, membrane curvature

## Abstract

Dysregulation of nuclear envelope (NE) assembly results in various cancers; for example, renal and some lung carcinomas ensue due to NE malformation. The NE is a dynamic membrane compartment and its completion during mitosis is a highly regulated process, but the detailed mechanism still remains incompletely understood. Previous studies have found that isolated diacylglycerol (DAG)-containing vesicles are essential for completing the fusion of the NE in nonsomatic cells. We investigated the impact of DAG depletion from the *cis*-Golgi in mammalian cells on NE reassembly. Using advanced electron microscopy, we observed an enriched DAG population of vesicles at the vicinity of the NE gaps of telophase mammalian cells. We applied a mini singlet oxygen generator-C1-domain tag that localized DAG-enriched vesicles at the perinuclear region, which suggested the existence of NE fusogenic vesicles. We quantified the impact of Golgi-DAG depletion by measuring the in situ NE rim curvature of the reforming NE. The rim curvature in these cells was significantly reduced compared with controls, which indicated a localized defect in NE morphology. Our novel results demonstrate the significance of the role of DAG from the *cis*-Golgi for the regulation of NE assembly.

The nuclear envelope (NE) is a subcompartment of the endoplasmic reticulum (ER) and it breaks down and reforms during an open mitosis. However, the detailed molecular mechanism regarding its reassembly remains to be determined. Although envelopment of the chromatin by the ER is one of the NE reassembly models (1) and contact between ER cisternae and chromosomes has been shown to initiate mammalian NE reassembly at anaphase (2), these descriptions do not explain the necessity of membrane fusion or the possible requirement of correctly formed curved regions for the localization of the nuclear pore complexes.

Proteins play an important role in membrane fusion and morphology (3–6), but these mechanisms also require lipids. Membrane fusion can be facilitated in a protein-free context (7, 8), meaning that lipids are critical effectors during the NE fusion.

To explain membrane fusions in NE completion, the vesicle fusion model provides an additional mechanism (9). Membrane fusion as well as membrane regions with high curvature, such as the NE rims, are promoted by the presence of negatively curved lipids, where diacylglycerol (DAG) destabilizes the localized membrane morphology (10–12). The most curved region of the NE, which exists between the inner and outer nuclear membranes where nuclear pore complexes are recruited, is referred to as the NE rim.

As mentioned above, neutral lipids, such as DAG, which have a high negative spontaneous curvature, locally destabilize the lamellar bilayer to induce membrane fusion (13, 14). The understanding of how this lipid behaves during membrane fusion has mostly resulted from in vitro or reconstitution experiments (10, 11, 15), and its complex behavior has not been directly examined in live cells or model organisms.

Our group has isolated a population of membrane vesicles known as MV1, a distinctive membrane compartment in vivo with atypical polyphosphoinositide compositions (16), as one of the critical NE precursors of echinoderm male pronuclei. MV1 has elevated levels of phosphoinositides, a lipid-modifying enzyme, PLCγ, and its upstream regulator, src family kinase 1 (SFK1) (16, 17). These three proteo-lipid components are the fusion “tool-kit” responsible for localized production of DAG.

In mammals, DAG localizes at the NE, ER, and Golgi (18). Using a rapamycin analog (rapalog)-based heterodimer system (19, 20), we have demonstrated the structural role of DAG in mammalian NE reassembly where localized depletion of DAG at the NE and ER results in failure of NE reassembly and abnormal ER morphology (18). The abnormal ER morphology and fragmented NE from these experiments resulted in taking our experiments forward to determine whether localized highly curved regions, such as the rim curvature of the NE, would be affected by the acute and inducible depletion of DAG. To examine this, we set out to investigate the impact of DAG depletion from the *cis*-Golgi, a reservoir of DAG in mammalian cells, on mammalian NE reassembly using the rapalog dimerization approach.

Here, we show that locally depleted DAG from the *cis*-Golgi impacts the NE rim curvature of the reforming NE. The rim curvature in these cells is significantly reduced compared with the controls. We measured the in situ rim curvature and the pore diameters to illustrate the localized defect in NE morphology. These membranous defects may result in affecting the proper nuclear pore insertions. Our results demonstrate the significance of the role of DAG from the *cis*-Golgi for the regulation of NE assembly.

## MATERIALS AND METHODS

### Cell line and maintenance

The HeLa cell line was obtained from the ATCC (ATCC #CCL2). The cells were grown in DMEM containing 10% FBS (Gibco) supplemented with penicillin (100 U/ml) and streptomycin (100 μg/ml) and incubated at 37°C in 10% CO_2_.

### Constructs

Golgi reassembly stacking protein 65 (GRASP65)-GFP and its non-Golgi-targeting mutation, GRASP65.G2A-GFP, were gifts from Professor Yan-Zhuang Wang (University of Michigan). GRASP65-GFP-2FKBP and GRASP65.G2A-GFP-2FKBP were created by adding a 2FKBP domain into the given constructs at its C terminus with flanking *Bsr*GI sites. The GRASP65, GFP tag, and 2FKBP rapalog-binding domain were made into such a sequence so that the constructs could successfully localize to the Golgi and recruit DAG kinase εK (DGKεK). GFP-2FKBP-LBR, RFP-Flag-FRB-DGKεK, and RFP-Flag-FRB-DGKεK.D434N were created as described in (18).

### Confocal imaging

Cells were grown on gridded glass coverslips in 3.5 cm dishes (MatTek Corporation) and double transfected with the rapalog dimerization constructs described above. Cells were transfected with 0.5 μg of the DNA of each construct using Lipofectamine LTX and PLUS reagent (Invitrogen) in OPTIMEM medium (Gibco BRL) as recommended by the manufacturer. The cells were left overnight in the transfection mix in antibiotic-free medium before replacing it with fresh medium. Experiments were performed 36–48 h post transfection. The transfected cells were stained with ER tracker Blue-White DPX (Invitrogen) at a final concentration of 1 μM according to manufacturer’s protocol before the rapalog experiment (18, 19). Cells were incubated in a 5 l/h humidified chamber with 10% CO_2_ adapted to a Zeiss confocal microscope (Zeiss LSM 710). Images were acquired prior to and after the recruitment of DAG kinase to the targeted membrane compartment, ∼30 min to 1 h after addition of 500 nM rapalog heterodimerizer (Clontech). Cells were then imaged every 30–45 min at low pixel resolution (512 × 512 with a 0.6× zoom) from interphase to late anaphase to limit laser damage. A high pixel resolution (1,024 × 1,024 with a 2× zoom) series of images was also acquired from late anaphase to cytokinesis.

Each experiment was performed at least three times. All images were treated in the same manner, i.e., only minor adjustments of brightness and contrast were applied to every pixel.

### Correlative light and electron microscopy

Live cells were followed to the required stage of mitosis using confocal microscopy. Cells were then fixed in 4% electron microscopy (EM) grade formaldehyde (TAAB) in 0.1 M phosphate buffer (PB) (pH 7.4) to halt the cell cycle prior to reimaging for bright-field and high-magnification fluorescence signals. Cells were subsequently located using the grid number of the gridded glass coverslip (MatTek). Secondary fixation was performed in 1.5% glutaraldehyde/2% formaldehyde in 0.1 M PB for 30–60 min. After fixation, coverslips were carefully removed from the MatTek dishes and washed several times in 0.1 M PB.

For transmission EM (TEM), the cells were postfixed in 1.5% potassium ferricyanide/1% osmium tetroxide for 1 h before rinsing in PB and incubating in 1% tannic acid in 0.05 M PB for 45 min to enhance membrane contrast. After a brief rinse in 1% sodium sulfate in 0.05 M PB, the coverslips were washed twice in distilled water, dehydrated through an ascending series of ethanol to 100% prior to infiltration with epoxy resin and polymerization overnight at 60°C. The coverslips were removed from the resin blocks by plunging briefly into liquid nitrogen. The cells of interest were identified by correlating the grid and cell pattern on the surface of the block with previously acquired confocal images. The area of interest was cut from the block and further trimmed by hand using a single edged razor blade to form a small trapezoid block face for serial ultrathin sectioning. Using a diamond knife, serial ultrathin sections of 70 nm thickness were cut through the entire extent of the cells of interest (80–140 sections) and collected on 1.5% Formvar-coated single slot grids. The sections were counterstained with lead citrate to further enhance contrast prior to viewing in the electron microscope (FEI Tecnai G2 Spirit BioTWIN with Gatan Orius CCD camera). Serial images were stacked and aligned, and the NE, ER, and centrioles and vesicles were manually segmented using Amira (FEI), based on their electron density and morphological features. Circular membrane structures with similar *x*, *y*, and *z* diameter were segmented as vesicles. Movies were created from 2D tiff stacks using Quicktime Player 7 Pro and compressed using Stomp (Shinywhitebox Ltd.).

For serial block-face scanning EM (SBF SEM), the fixed cells were processed following the method of the National Centre for Microscopy and Imaging Research (21), which impregnates the sample with high concentrations of heavy metals to introduce maximal contrast and conductivity when viewed in the scanning electron microscope. The cell of interest was identified by correlation of grid reference with previously acquired confocal images; this area was trimmed to a small trapezoid, excised from the resin block, and attached to a SBF SEM specimen holder using conductive epoxy resin (Circuitworks; CW2400). Prior to commencement of a SBF SEM imaging run, the sample was coated with a 2 nm layer of platinum to further enhance conductivity.

SBF SEM data were collected using a 3View2XP (Gatan, Pleasanton, CA) attached to a scanning electron microscope (Zeiss, Cambridge). To relocate the cell of interest in the scanning electron microscope, an overview was first acquired at 5 kV, sufficient to penetrate the platinum coating and generate an image of the underlying sample. Inverted backscattered electron images were then acquired through the entire extent of the cell of interest at a resolution of 8,192 × 8,192 pixels (horizontal frame width of 36.74 μm; pixel size of 4.5 nm) using a 2 μs dwell time and 50 nm slice thickness. The scanning electron microscope was operated in variable pressure mode at 5–10 Pa, with high current mode active, 20 μm aperture, an accelerating voltage of 2 kV, and an indicated magnification of 7,000. Typically, around 400 slices were necessary to image an entire cell, representing a total volume of approximately 27,000 μm^3^.

As data were collected in variable pressure mode, only minor adjustments in image alignment were needed, particularly where the field of view was altered in order to track the cell of interest.

### Electron tomography

For electron tomography (ET), samples were prepared as detailed above, but 200 nm-thick serial sections were collected through the entire extent of the cells of interest. Tomograms were acquired from the 200 nm sections at targeted regions in the reforming NE, either at specific gaps or where collections of vesicles were evident in close proximity to the reforming envelope. Images were collected at 1° intervals across a maximal tilt range of ±70°, with 0.79 nm width per pixel for 2,048 × 2,048 pixels, with a per pixel resolution of 0.79 nm. Tomograms were processed with IMOD (22), using patch tracking for alignment and simultaneous iterative reconstruction technique for volume reconstruction. The reconstructed volume was exported as a series of 2D tiff images, and the NE and adjacent membranous structures, including vesicles, were manually segmented and reconstructed using Amira (Visage Imaging, Berlin). Movies were created from the 2D tiff stacks using Quicktime Player 7 Pro, and compressed using Stomp (Shinywhitebox Ltd.). It is of note that one cannot negate embedding artifacts enough to have a definitive measurement of curvature and so cryo-microscopy is preferred; however, the cryo-tomography required is extremely technically challenging and cannot currently be performed for rare correlative events due to the complexity of sample preparation and difficulties in targeting specific cells through the cryo workflow. It should be noted that if this methodology were to be used for a larger study, or under varying imaging conditions, there would be a justification to verify these trends in a case-by-case manner.

### Segmentation of ultrastructure

Serial micrographs were stacked and aligned using Amira (Visage Imaging, Berlin). The pixels of NE, ER, centrioles, and vesicles were manually traced based on their electron density and morphological features on the micrograph. Circular membrane structures with similar *xy* and *z* diameter were segmented as vesicles in the TEM analysis of the telophase cell. Discontinuities of the reforming NE around the chromatin were segmented as the NE gaps.

### Mini singlet oxygen generator photo-oxidation

Cells were transfected with GFP-PKCεC1aC1b-SOG and light microscopy was performed to identify cells of interest prior to initial fixation with 4% formaldehyde in 0.1 M PB, and secondary fixation with 2.5% glutaraldehyde in 0.1 M PB. The cells were washed in 0.1 M PB and incubated in blocking buffer (50 mM glycine, 10 mM KCN, and 20 mM aminotriazole in 0.1 M PB) for 30 min. The dishes were then loaded into a customized cooling chamber to maintain the samples at ∼4°C. The lid of the chamber was connected to an oxygen supply at a rate of 2.5 l/min. The cells were then incubated in diaminobenzidene solution (5.4 mg in 10 ml 0.1 M PB) and photo-oxidized for 30 min using a Hg lamp, after which they were further fixed on ice with 2% formaldehyde/1.5% glutaraldehyde in 0.1 M PB for 30 min, and postfixed with 0.5% osmium tetroxide in 0.1 M PB for 30 min before embedding as described above for correlative light microscopy and EM. The photo-oxidized cells were relocated using the MatTek grid coordinates and serially sectioned for examination using TEM.

### NE rim curvature measurements

Blocks, stained as per the serial block-face method, were instead serial sectioned at 300 nm onto Pioloform-coated slot grids. Fifteen nanometer gold particles (Aurion) were drop cast for 5 min on both sides with the excess blotted off. Micrographs were acquired through the cell and two areas selected on each of the daughter cells. Single tilt tomograms were taken of the selected areas using a 200 KeV transmission electron microscope (T20; FEI) with a LaB_6_ filament and a 4k × 4k Eagle (FEI) bottom mounted camera. The tilt series was taken, using the FEI software, at 2° intervals between ±60° where possible, at a nominal magnification of 14,500×, giving pixel sizes of 0.74 × 0.74 nm^2^. The tilt series were reconstructed using IMOD (22), with subsequent image analysis using Fiji (23) and statistical analysis using Prism (GraphPad).

Tortuosity of the nuclear membranes could not be analyzed quantifiably from the 3D tilt series reconstructions due to the volumetric field of view. Instead, if the membrane curvature has been disrupted, a requirement for pathological tortuosity, it would be expected to be evident at the tight curvatures between the inner and outer membranes, defined here as rim diameter, to be consistent with modeling of the ER (24).

To achieve an estimate of any altered curvature, it was decided to quantify the nuclear pores. Two measures were taken (Fig. 4): the diameter of the pore and the distance between the inner and outer nuclear membrane, its rim diameter. Initially, to achieve good membrane contrast, the 3D reconstructed image stacks were reduced by binning by a factor of four (leaving 3 × 3 × 3 nm^3^ voxels in *x-y* and *z* directions), and dynamic range to 8 bit after suitable windowing. Three-dimensional image clips were taken from every nuclear pore completely enclosed in the reconstruction, as described by Fig. 4. Each clip was rotated such that the membranes are parallel to the *x* axis, then rotated around the *x* axis by 10° increments until the pore appeared (by eye) as near to a circle as possible. From this point, the clip was rotated back by 90°, making the pore perpendicular to the viewed plan. The width and rim diameter were then measured at a depth of the widest diameter of the pore by measuring the distance between centers of the dark bilayer membranes where they just become parallel.

## RESULTS

### The presence of vesicles in the vicinity of the reforming NE

In our previous work on the echinoderm model, we showed that MV1, a vesicular compartment enriched in PLCγ and phosphoinositides, was recruited to each stage of membrane fusion required for NE and zygote formation (17). Based on these results, we predicted that vesicles enriched in phosphoinositides or their derivative, DAG, might also play a role in NE assembly in mammalian cells.

**Fig. 1.**
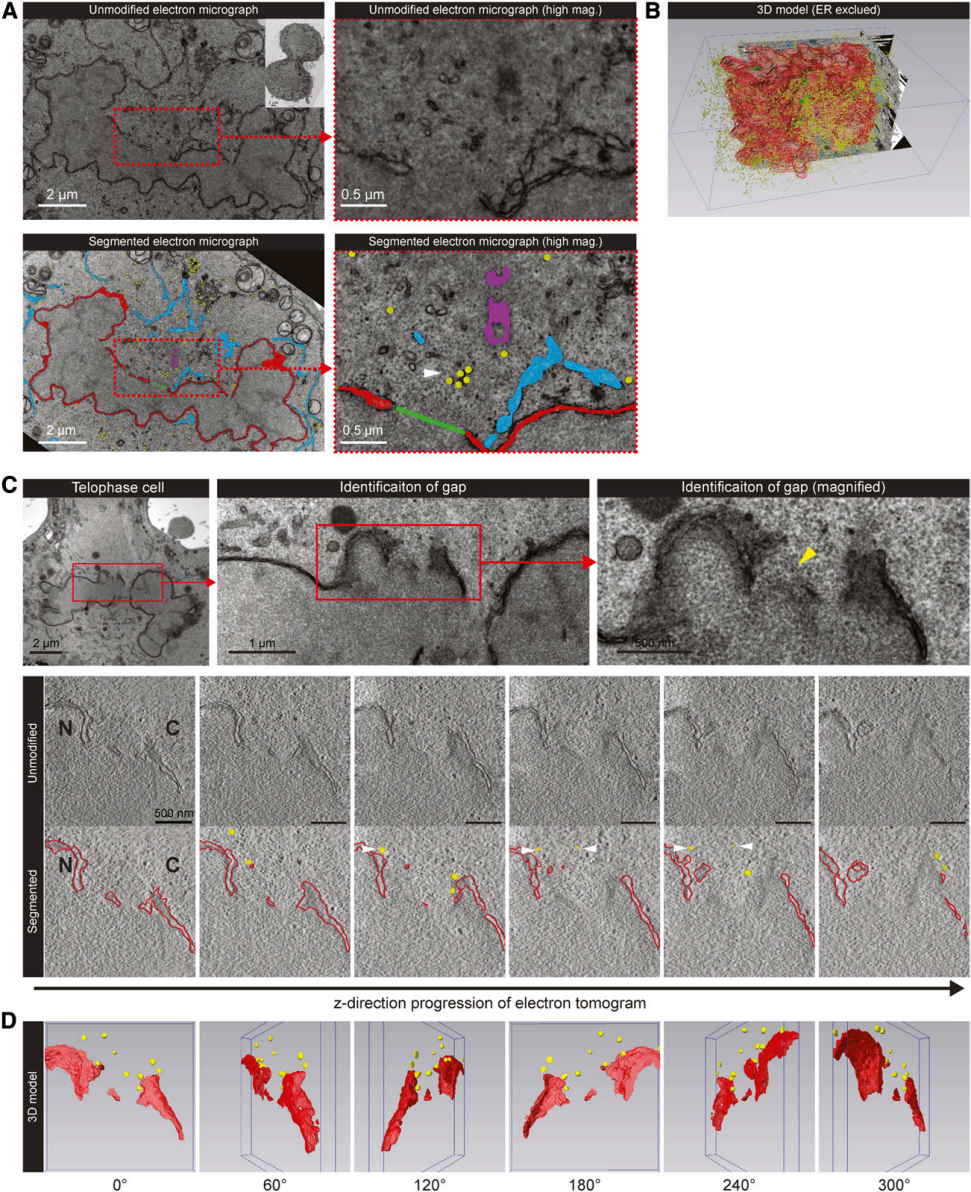
Detection of perinuclear vesicles by TEM and ET. A: A HeLa cell was fixed at telophase (inset), cut into serial sections, and prepared for TEM analysis. Cellular structures and gaps were traced manually (segmentation) in different colors: NE (red), ER (cyan), centriole (purple), vesicles (yellow), gaps (green). The top and bottom panels depict the unmodified and segmented electron micrographs, respectively. The white arrow indicates the vesicles close to a NE gap. B: A 3D model displaying all segmented structures described in A except the ER. A large number of vesicles (n = ∼14,000) was detected in the telophase cell. C: Top panels: Electron micrographs from a 200 nm-thick section taken through a telophase HeLa cell. The identified gap (yellow arrow) was analyzed by ET to produce a tomogram with a thickness of 163 nm. Bottom panels: Snapshots of the unmodified and segmented tomogram along the *z*-axis, with the reforming NE segmented in red and vesicles segmented in yellow. White arrows indicate virtual space occupied by the vesicles. N, nucleus; C, cytoplasm. D: Three-dimensional model reconstructed from the segmented tomogram at different angles. Scale bars, as indicated.

To demonstrate the presence of vesicles that might participate in NE completion at late mitotic stages, we investigated the ultrastructure of telophase cells by TEM. **Figure 1A** and C show that multiple gaps existed at the NE of the examined telophase cells, and vesicles were detected in the proximity of these gaps. ET confirmed that these membrane structures occupied a spherical space in the virtual volume of the tomogram (Fig. 1C, supplemental Movie S1), implying the presence of vesicles as opposed to tubules. Some vesicles were very close to the edge of the reforming NE, as indicated by the reconstructed 3D model (Fig. 1D). However, there were numerous vesicles in a telophase cell. A comprehensive 3D reconstruction revealed at least 14,000 vesicles in one of the daughter cells at this mitotic stage (Fig. 1B). Therefore, we focused on studying the vesicles that were close (500 nm) to the reforming NE because physical contact is required for membrane fusion.

### The number of vesicles is significantly higher at the large gaps

**Fig. 2.**
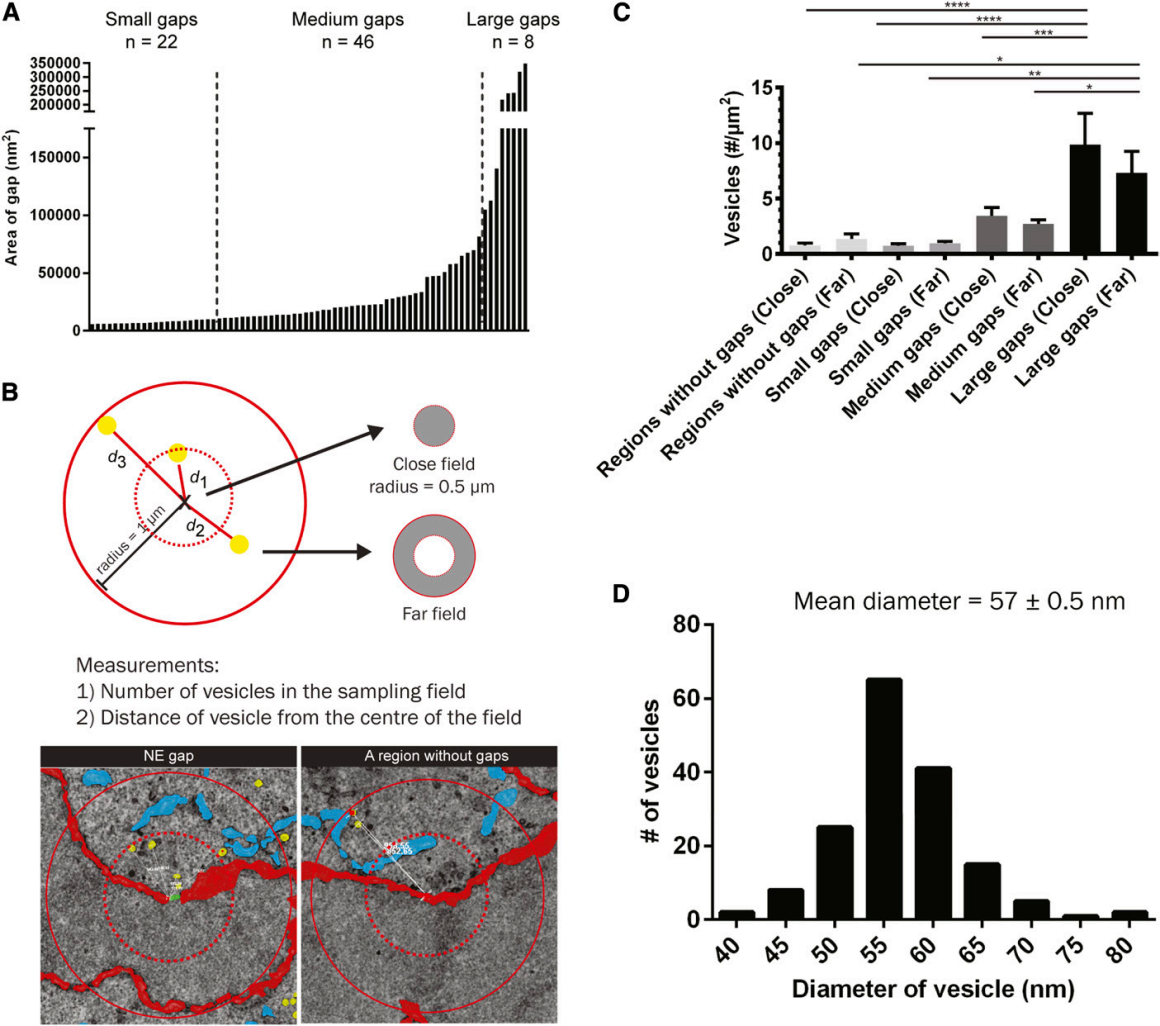
Quantitative analysis of gaps and vesicles at the NE of telophase cells. A: There were 76 gaps at the NE of the cell described in Fig. 1A. The gaps were categorized based on their size. Small (n = 22), medium (n = 46), and large gaps (n = 8) differed in their area by an order of magnitude. B: A circular sampling field with a radius of 1 μm was drawn, the center of the sampling field is either an end point of a gap or a random point at the NE as a control (n = 10). In the field, the number of vesicles and their distance from the center were measured. The field was subdivided into close and far fields as indicated. C: The number of vesicles per area analyzed by one-way ANOVA. The number of vesicles in the close and far fields of the large gaps was significantly higher than that in other regions. *P ≤ 0.05, **P ≤ 0.01, ***P ≤ 0.001, ****P ≤ 0.0001. D: The diameter of vesicles plotted into a histogram.

To understand how vesicles distributed around the reforming NE of a telophase cell, we compared the concentration of vesicles at the regions with gaps and the regions without gaps (**Fig. 2**). A gap was defined by the shortest distance between the two converging edges of the reforming NE in the *x*-direction multiplied by its thickness in the *z*-direction, not exceeding 140 nm. The size range of the gaps varied within three orders of magnitude; therefore, we grouped the gaps into small (5,800–9,900 nm^2^), medium (11,000–82,000 nm^2^), and large (100,000–350,000 nm^2^), based on their vertical area (*x-z*) in the micrographs (Fig. 2A). A cylindrical volume was defined as the “vicinity” of different regions of interest and the volume has a radius of 1 μm so that it did not exceed the boundary of the cell. For a gap, one of its ends was selected as the center of the cylindrical volume (Fig. 2B). We compared the concentration of the vesicles in the vicinity with the same volume at different regions. The vesicle concentration at the large-gap regions was significantly higher than that in other regions; whereas at medium-gap-regions and small-gap-regions, the vesicle concentration reduced in relation to the gap size (Fig. 2C). This might imply that the vesicles in the large-gap regions were being utilized to induce membrane fusion of the NE fragments. The mean diameter of all the vesicles in the close field was 57 ± 0.5 nm (Fig. 2D).

### DAG-enriched vesicles in the proximity of the NE

**Fig. 3.**
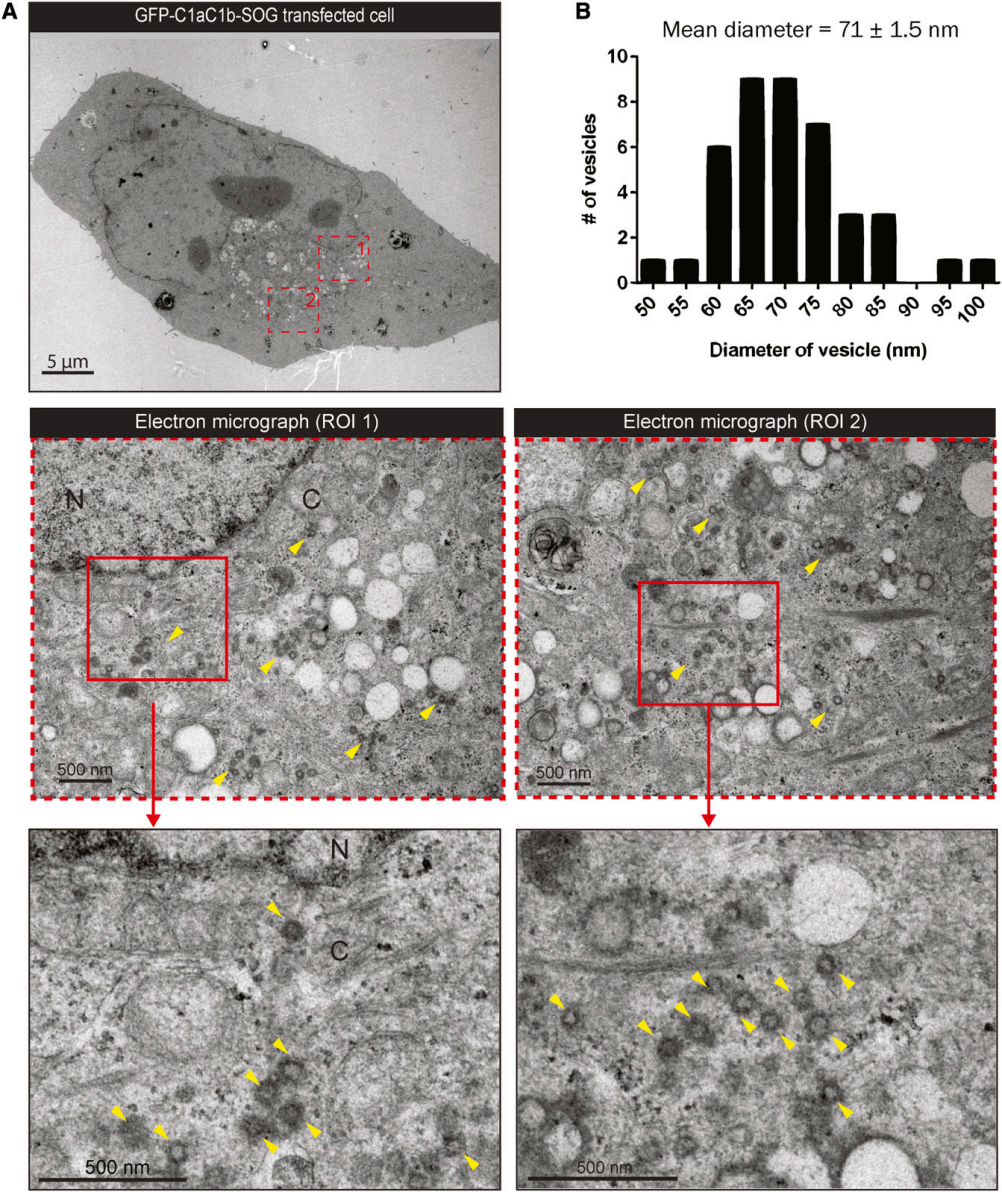
DAG-enriched vesicles at the NE of interphase cells. A: Electron micrographs of an interphase HeLa cell transfected with the DAG reporter (GFP-C1aC1b-SOG) for 16 h. The transfected cell was fixed and photo-oxidized in the presence of DAB to enhance local contrast during EM sample preparation. Red dashed boxes corresponding to the magnified regions of interest (ROI). In the electron micrographs, numerous vesicles had enhanced electron density and were distinguished from the background EM contrast (yellow arrows). N, nucleus; C, cytoplasm. Scale bars, as indicated. B: The diameter of miniSOG-labeled vesicles in the proximity of the NE (within a radius of 1 μm, as described in Fig. 2B) was measured as described in Fig. 2D and plotted into a histogram. The mean diameter of these vesicles was 71 nm and most of the vesicles (n = 18) fell into the 65 and 70 nm group.

To identify DAG-enriched vesicles, we exploited the C1a-C1b domain of PKCε and its nonbinding mutant, C1aC1b-W264G. This domain specifically recognizes the subcellular localization of DAG. To illustrate the DAG specificity, the nonbinding mutant was utilized (supplemental Fig. S2). Moreover, to enable the direct recognition of these vesicles at the EM level at the vicinity of the NE, a mini singlet oxygen generator (miniSOG) approach was utilized. The conventional methods (e.g., immunogold) for TEM were inappropriate because the permeabilization step extracted the lipids of interest. The miniSOG tag (25) provided TEM contrast via photo-oxidation. This step released oxygen to polymerize 3,3′-diaminobenzidine (DAB) at the targeted structures, which attracted preferential osmium staining. **Figure 3A** shows that many vesicles were labeled with GFP-PKCεC1aC1b-SOG reaction products in an interphase cell at the perinuclear region. The mean size of the miniSOG-labeled vesicles was greater due to the formation of the DAB polymer (Fig. 3B). Hence, this demonstrated that the C1a-C1b domain recognized the DAG-enriched vesicles. If the C1 domain did not bind to DAG, the size of the vesicles would remain unchanged. We suggest that these DAG-enriched vesicles are present at interphase for the maintenance of the NE morphology.

### Golgi-DAG depletion by the rapalog dimerization system

To investigate the localized and inducible impact of Golgi-DAG depletion on NE reassembly during mitosis, we used the rapalog dimerization system and the Golgi-targeting domain of GRASP65, a *cis*-Golgi marker expected to be corecruited with DGKεK, providing spatial specificity. The C1 domain of DAG kinase specifically targets DAG (18).

Cells were transfected with the GRASP-GFP-2FKBP and RFP-Flag-FRB-DGKεK. Confocal images were acquired prior to and after the recruitment of DGKεK to the targeted membrane compartment, ∼30 min to 1 h after addition of 500 nM rapalog heterodimerizer. Cells were imaged every 30–45 min from interphase to late anaphase to limit laser damage. A series of high-resolution images were also acquired from late anaphase to cytokinesis. They were fixed after reaching late telophase, where the NE should be fully assembled.

**Fig. 4.**
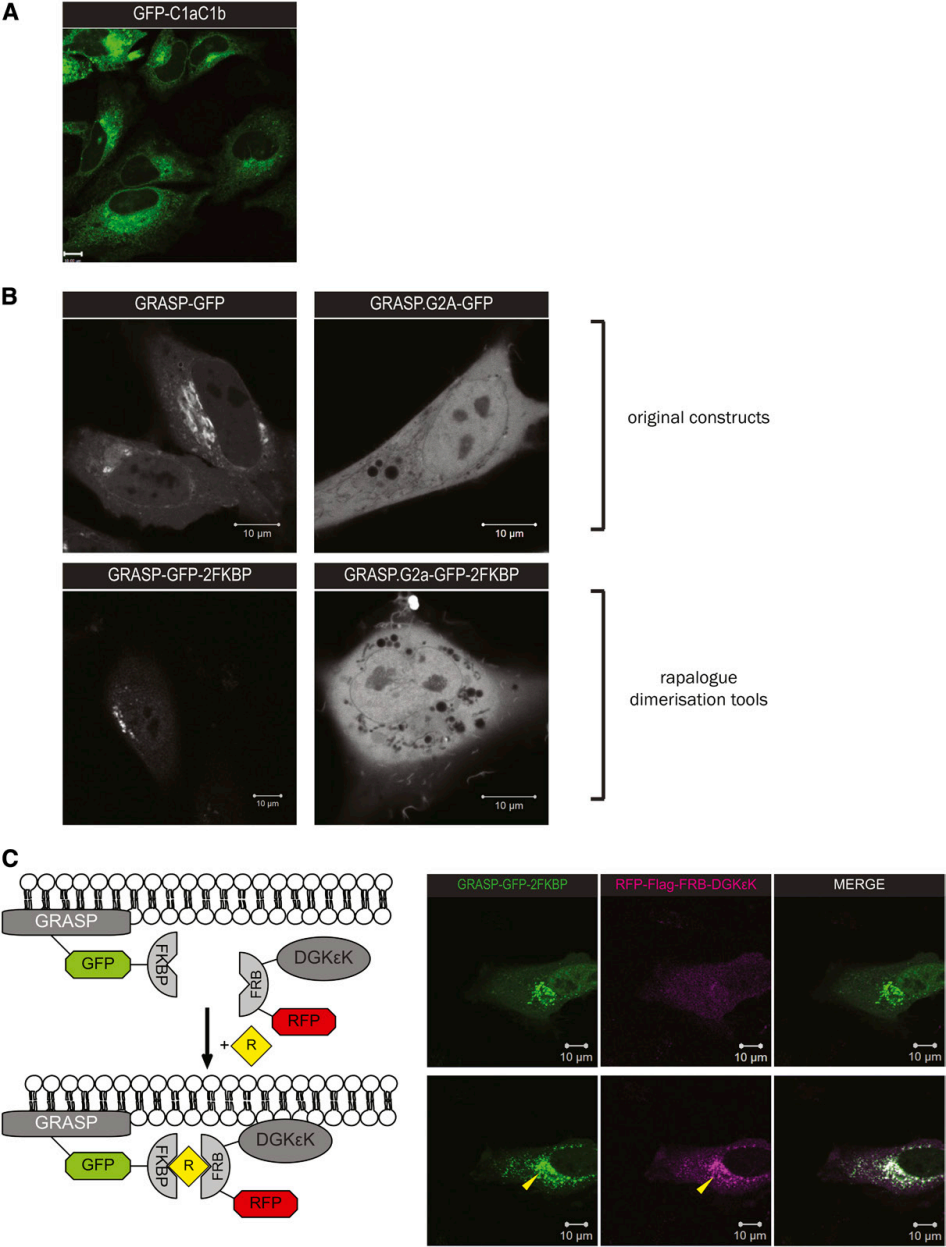
The rapalog dimerization system that targets the Golgi. A: DAG detection by GFP-PKCε^C1aC1b^. B: Localization of the original GRASP65 constructs and its rapalog dimerization unit-conjugated counterparts. GRASP65.G2A is a non-Golgi-binding mutation. C: Left: Diagram of the rapalog dimerization constructs that target DAG at the Golgi. Upon addition of rapalog, RFP-Flag-FRB-DGKεK (DGKεK), a lipid-modifying enzyme, is expected to be recruited to GRASP-GFP-2FKBP (Golgi) through the formation of a RFP-FKBP heterodimer so that DAG at the Golgi will be converted to phosphatidic acid (PtdOH). Right: Confocal images showing the successful recruitment of DGKεK (magenta) to the Golgi (green). Yellow arrows indicate the Golgi localization of both constructs 2 h post-rapalog addition.

DAG can be observed at the Golgi in addition to the NE and ER (**Fig. 4A**). Figure 4B shows that the rapalog construct, GRASP-GFP-2FKBP, has a similar localization compared with the original construct, GRASP-GFP, and the reported non-Golgi-binding mutation (G2A) indicated that the GRASP domain is Golgi-specific. Figure 4C indicates the successful recruitment of DGKεK to the Golgi when the rapalog was added.

**Fig. 5.**
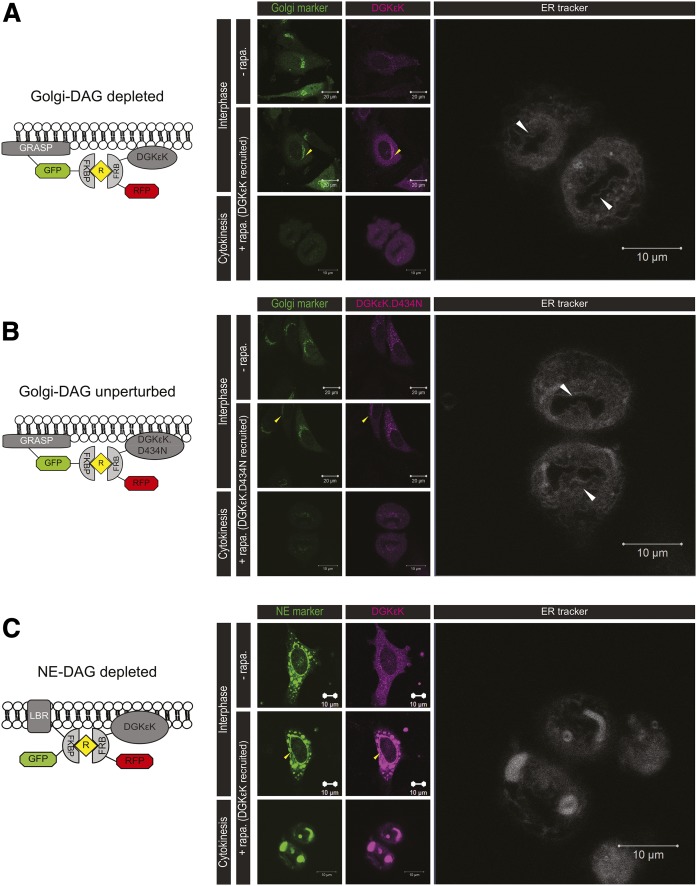
Morphology of a Golgi-DAG-depleted cell at light microscopy level. HeLa cells were double transfected with different rapalog dimerization constructs, stained with the ER tracker, treated with rapalog, and followed through mitosis before fixation at cytokinesis. Yellow arrows indicate the recruitment of the lipid-modifying enzymes to different cellular compartments. A: DGKεK was recruited to the Golgi in the presence of rapalog (Golgi-DAG depleted). The NE reformed around the chromatin as indicated by the ER tracker (white arrows). B: A catalytically inactive DGKεK (DGKεK.D434N) was recruited to the Golgi upon rapalog addition (Golgi-DAG unperturbed). The NE reformed around the chromatin (white arrows). C: DGKεK was recruited to the NE in the presence of rapalog (NE-DAG depleted). There was no obvious NE reformed.

To examine the influence of DAG depletion on the formation of the NE at the late mitotic stages, the cells were fixed at late telophase/cytokinesis. **Figure 5** shows the interventions by the rapalog dimerization system imaged by fluorescence microscopy. DGKεK and its catalytically inactive mutant, DGKεK.D434N, were recruited to the Golgi upon rapalog addition (Fig. 5A, B). ER tracker Blue-White DPX functions as the marker to determine whether the NE was reformed. In both Golgi-DAG-depleted (DGKεK recruited) and Golgi-DAG-unperturbed conditions (DGKεK.D434N recruited), signals of the ER tracker were observed around the chromatin. Unlike the ER/NE-DAG depletion (Fig. 5C), where the NE was totally fragmented at the fluorescence microscopy level (18), an obvious change here was not perceived. This was expected, as the depletion of DAG from the ER would affect the NE morphology prominently, as NE is a subunit of the ER.

To observe whether a minor fragmentation occurred at the electronic resolution, the complete 3D NE ultrastructure in Golgi-DAG-depleted and Golgi-DAG-unperturbed cells was examined by SBF SEM, and again only a mild or no NE fragmentation was observed in both conditions (supplemental Fig. S1).

We suggest that Golgi-DAG depletion was insufficient to cause a general NE morphological defect. However, this would not rule out the effect Golgi-DAG depletion may have on the finer structures of the NE. We therefore postulated that there would be localized morphological defects at high curvature regions of the NE, where DAG may have a localized role in maintaining these high curvature regions.

### Golgi-DAG depletion causes a decrease in NE rim curvature

To test this postulation, we measured, using a higher resolution imaging technique, the most curved region of the NE, which exists between the inner and outer nuclear membranes where nuclear pore complexes are recruited. This region is referred to as the NE rim. The NE rim curvature was utilized to determine the effect of the Golgi-DAG depletion on localized membrane curvature (24).

Four experimental conditions were compared: DAG kinase with rapalog (DKGεK+Rap), DAG kinase mutant with rapalog (D434N+Rap), DAG kinase without rapalog (DKGεK-Rap), and untransfected cells. From these four conditions, nuclear pores from 21 tomograms were sampled evenly across the NE of telophase cells and measurements were acquired under these conditions. Beam-induced mass loss causes anisotropic section shrinkage, usually thought of as approximately 30% in thickness and 2–8% in *x*-*y*. To ensure that our measurements were not biased by the anisotropy of section shrinkage in ET, samples with the most pores from four tomograms (DKGεK+Rap) were initially tested for dependence on the pore’s angle in the sample. We determined that there was no significant dependence (*P* = 0.089) on the rotated angle to rim curvature measurement, but there was a dependence (*P* = 0.046) on pore diameter to the angle (Pearson, data not shown). This is likely a low dependence due to the hard resin used and not using excessive beam intensity. Nevertheless, the trend in the measurements was well below the innate variation and, therefore, considered as insignificant for further analysis.

**Fig. 6.**
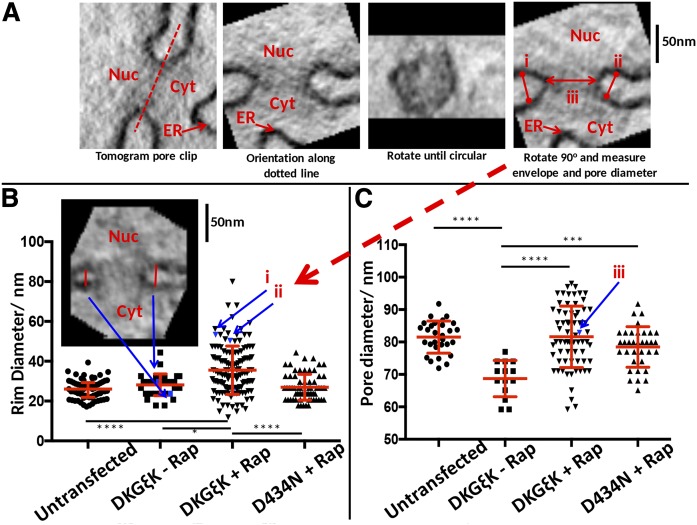
NE rim curvature is affected by the lack of DAG at the Golgi. A: The analysis sequence from a nuclear pore 3D image clip (see supplemental Fig. S2 for whole tomographic reconstruction). The image clip is rotated until the nuclear pore is parallel to the *x*-axis, i.e., along the red dotted line between the nucleus (Nuc) and cytoplasm (Cyt). The image clip is then rotated around the *x*-axis until the most circular pore can be observed, followed by a 90° rotation back to achieve a pore perpendicular to the field of view. Rim diameters (i and ii) and pore diameter (iii) are measured and presented as in B and C, respectively. B: The rim diameters for control HeLa cells (untransfected, two pairs of cells), DKGεK-transfected but without a rapalog (DKGεK-Rap, one pair of cells), DKGεK-transfected with rapalog (DKGεK+Rap, two pairs of cells), and D434N transfected with rapalog (D434N+Rap, two pairs of cells). The red lines indicate the median ± interquartile range. The analysis was Kruskal-Wallis test with Dunn’s multiple comparison test. C: Pore diameters for the same pores analyzed in B. The red lines indicate the mean ± standard deviation. The analysis was one-way ANOVA with Tukey’s multiple comparisons test. For B and C, the asterisks indicate significance (*P < 0.05, ** *P* < 0.001, ****P < 0.0001); all other combinations were not significant.

The rim diameters measured from where the inner and outer membranes become parallel were quantified (**Fig. 6A**, B), and we determined that the four groups did not all fit a normal distribution. Therefore, a Kruskal-Wallis with Dunn’s multiple comparison test was performed. There was an indication that the DKGεK+Rap rim diameter measurements had a bimodal distribution. The rim diameter was significantly different only in comparison with DKGεK+Rap, indicating the principle of our measurement criteria was valid and useful. This is indicative of the alteration of membrane curvature in the localized absence of DAG.

It was observed, but not quantified, that the DKGεK+Rap seemed to have an anomalous rim diameter change, with both the rim curvatures and the pores not in the plane of the envelope as normally observed.

The alignment of nuclear pores at the rim of the NE can be a parameter for NE defects if the analysis was performed on a larger scale. The rim diameters were converted into curvature (κ) by κ = 2/RD. The median ± interquartile range/2 for κ_untransfected_ = (0.077 ± 0.010) nm^−1^, but was significantly reduced (*P* < 0.0001) for the Golgi-DAG-depleted cells where κ_DKGεK+Rap_ = (0.060 ± 0.014) nm^−1^. This demonstrated that without the DAG from the Golgi, a tight localized curvature was not formed.

The pore diameter measurements (Fig. 6C) fitted a normal distribution. Therefore, a one-way ANOVA analysis with Tukey’s multiple comparisons test was performed to determine the statistical significance. The curvature of the pore ring is far less than the rim diameter and only the DKGεK-Rap gave a significant difference in pore diameter (Fig. 6C). It is difficult to propose what may have caused this. Perhaps it was due to experimental rather than biochemical differences.

The four tomograms were taken in the same session under identical conditions, marking the highly significant difference of the rim curvatures within DKGεK+Rap. It was unlikely that the other conditions produced different measurements due to an artifact, as the conditions were kept as similar as possible.

In summary, this new quantitative method of measurements provides in situ membrane curvature values. It is by using such a quantitative method that we demonstrated a new concept that the tight rim curvature of the NE requires DAG that is targeted from the Golgi to the gaps during NE formation.

## DISCUSSION

Membrane vesicles have been isolated as NE precursors from several nonmammalian cell-free assays decades ago (26–28). These vesicles have been shown to constitute a specific cellular compartment enriched in membrane fusion machinery in situ (16, 17). Nevertheless, the existence of vesicular NE precursor in mammals is unknown. Using TEM and ET, we have detected vesicles in the vicinity of the reforming NE in telophase cells (Fig. 1), and the concentration of these vesicles is significantly higher in the large-gap regions than in regions of smaller gaps and regions without gaps. Such an observation suggests that vesicles play a role at the gaps for NE completion (Fig. 2). Moreover, it is unlikely that completion of the NE is solely contributed by the ER cisternae.

In the echinoderm NE assembly assay, Golgi-derived vesicles are required for recruiting the ER and completing the NE of male pronuclei through membrane fusion (29). In mammals, there is evidence for the presence of DAG (30) and its precursor, PtdIns(4,5)P_2_ (18, 31), at the Golgi. A recent study has shown that PLCγ1, which hydrolyzes PtdIns(4,5)P_2_ to DAG, participates in Golgi-DAG production (32). Furthermore, Golgi membranes (vesicles and cisternae) are detected in the perinuclear region during late mitotic stages (33, 34). These findings are reminiscent of the fusion machinery-enriched vesicles needed for NE reassembly in vitro (17) and the perinuclear vesicles detected in this study.

We applied GFP-miniSOG to visualize DAG in fluorescence microscopy and EM. This is the first study to access the usefulness of miniSOG constructs in labeling small structures such as vesicles. Our results indicate the presence of DAG-enriched vesicles in the cytoplasm of interphase cells, and some of these vesicles localized in the perinuclear region (Fig. 3). We suggest that these vesicles are required for the maintenance of the NE morphology and perhaps would participate in NE completion in later mitotic stages.

When we recruited high levels of DGKεK to the Golgi, the newly formed NE was not grossly fragmented and the quantification of the number of gaps created due to the lack of DAG at the Golgi was inconclusive. The catalytically inactive DGKεK did not cause any observable phenotype (supplemental Fig. S1).

To test whether the lack of DAG at the Golgi affected a more definite structure of the NE, we developed a novel quantitative method for measuring in situ curvature of the NE rim curvature. The results showed that the rim curvature in the absence of DAG from the Golgi was significantly lower than in its presence. The catalytically inactive DGKεK did not affect the rim curvature. The curvature of the NE pore is far less than the NE rim diameter, so one would not expect it to be as affected by DKGεK alteration, and this is borne out in the results. We have demonstrated a suitable quantitative methodology for determining curvature alteration, which, in turn, could be linked to the pore complex binding to the NE rim. Our current methodology has limitations due to dehydration artifact in the EM processing causing more tortuous membranes. The main result is that measurements have more variation, so if anything, would make significance harder to achieve. This error could be reduced, if necessary, by cryo-techniques such as high pressure freezing and freeze substitution of the aqueous component.

Our investigations have now provided evidence for the regulatory role(s) of both NE- (18) and Golgi-DAG during mitosis. A follow-up question is: How are these vesicles targeted to the gaps in such a manner that would deliver the required localized DAG for maintaining the appropriate NE rim curvature?

The presence of chromatin-targeting proteins, such as LBR, in NE precursors has been reported (35, 36), and this could provide a mechanism. However, the perinuclear vesicles have a small diameter of ∼55 nm, as well as constrained negative curvature, and, therefore, may not have sufficient surface for binding receptors. We speculate that membrane tethering, recruitment of membrane to a specific location by peripheral membrane proteins (tethers), is a mechanism to recruit vesicles to the gaps. Many tethers are originated from the Golgi (37), among them the p115 that has been reported as a general fusion factor interacting with other Golgi proteins such as giantin, GM130, and GRASP65 (38, 39). The trafficking direction and mechanism of p115-dependent tethering remains controversial, but it is notable that p115 has a capacity to bind to various targets (40). Interestingly, p115 does not bind to mitotic Golgi (41), giving us a hint that p115 may interact with NE/ER proteins and recruit Golgi vesicles to the reforming NE. Moreover, membrane curvature sensors may assist the recruitment of vesicles to the gaps. For instance, munc13 has a C1 domain targeting DAG and has been reported as regulating membrane fusion in different compartments (42–44).

Overall, in this study, we have developed a quantitative methodology that has permitted us to address the importance of the DAG localized at the *cis*-Golgi and how it may be implicated in maintaining and “fine-tuning” the morphology of the NE in mammalian mitosis.

## Supplementary Material

Supplemental Data

Supplemental Data

## References

[1] Anderson D. J., and Hetzer M. W. 2008 Reshaping of the endoplasmic reticulum limits the rate for nuclear envelope formation. J. Cell Biol. 182: 911–924. 18779370 10.1083/jcb.200805140 PMC2528577

[2] Lu L., Ladinsky M. S., and Kirchhausen T. 2011 Formation of the postmitotic nuclear envelope from extended ER cisternae precedes nuclear pore assembly. J. Cell Biol. 194: 425–440. 21825076 10.1083/jcb.201012063 PMC3153650

[3] Martens S., and McMahon H. T. 2008 Mechanisms of membrane fusion: disparate players and common principles. Nat. Rev. Mol. Cell Biol. 9: 543–556. 18496517 10.1038/nrm2417

[4] Wickner W., and Schekman R. 2008 Membrane fusion. Nat. Struct. Mol. Biol. 15: 658–664. 18618939 10.1038/nsmb.1451 PMC2488960

[5] Fanaei M., Monk P. N., and Partridge L. J. 2011 The role of tetraspanins in fusion. Biochem. Soc. Trans. 39: 524–528. 21428932 10.1042/BST0390524

[6] Dumas F., Byrne R. D., Vincent B., Hobday T. M., Poccia D. L., and Larijani B. 2010 Spatial regulation of membrane fusion controlled by modification of phosphoinositides. PLoS One. 5: e12208. 20808914 10.1371/journal.pone.0012208 PMC2923163

[7] Chernomordik L., Kozlov M. M., and Zimmerberg J. 1995 Lipids in biological membrane fusion. J. Membr. Biol. 146: 1–14. 7563032 10.1007/BF00232676

[8] Larijani B., and Dufourc E. J. 2006 Polyunsaturated phosphatidylinositol and diacylglycerol substantially modify the fluidity and polymorphism of biomembranes: a solid-state deuterium NMR study. Lipids. 41: 925–932. 17180880 10.1007/s11745-006-5045-2

[9] Larijani B., and Poccia D. L. 2009 Nuclear envelope formation: mind the gaps. Annu. Rev. Biophys. 38: 107–124. 19416062 10.1146/annurev.biophys.050708.133625

[10] Das S., and Rand R. P. 1984 Diacylglycerol causes major structural transitions in phospholipid bilayer membranes. Biochem. Biophys. Res. Commun. 124: 491–496. 6541910 10.1016/0006-291x(84)91580-8

[11] Das S., and Rand R. P. 1986 Modification by diacylglycerol of the structure and interaction of various phospholipid bilayer membranes. Biochemistry. 25: 2882–2889. 3718927 10.1021/bi00358a022

[12] Zhendre V., Grelard A., Garnier-Lhomme M., Buchoux S., Larijani B., and Dufourc E. J. 2011 Key role of polyphosphoinositides in dynamics of fusogenic nuclear membrane vesicles. PLoS One. 6: e23859. 21931619 10.1371/journal.pone.0023859 PMC3169559

[13] Goñi F. M. 2014 The basic structure and dynamics of cell membranes: an update of the Singer-Nicolson model. Biochim. Biophys. Acta. 1838: 1467–1476. 24440423 10.1016/j.bbamem.2014.01.006

[14] Larijani B., Hamati F., Kundu A., Chung G. C., Domart M. C., Collinson L., and Poccia D. L. 2014 Principle of duality in phospholipids: regulators of membrane morphology and dynamics. Biochem. Soc. Trans. 42: 1335–1342. 25233412 10.1042/BST20140224

[15] Huang W., Jiang D., Wang X., Wang K., Sims C. E., Allbritton N. L., and Zhang Q. 2011 Kinetic analysis of PI3K reactions with fluorescent PIP2 derivatives. Anal. Bioanal. Chem. 401: 1881–1888. 21789487 10.1007/s00216-011-5257-z PMC3311999

[16] Byrne R. D., Veeriah S., Applebee C. J., and Larijani B. 2014 Conservation of proteo-lipid nuclear membrane fusion machinery during early embryogenesis. Nucleus. 5: 441–448. 25482196 10.4161/nucl.34422 PMC4164486

[17] Byrne R. D., Garnier-Lhomme M., Han K., Dowicki M., Michael N., Totty N., Zhendre V., Cho A., Pettitt T. R., Wakelam M. J., 2007 PLCgamma is enriched on poly-phosphoinositide-rich vesicles to control nuclear envelope assembly. Cell. Signal. 19: 913–922. 17184973 10.1016/j.cellsig.2006.10.011

[18] Domart M. C., Hobday T. M., Peddie C. J., Chung G. H., Wang A., Yeh K., Jethwa N., Zhang Q., Wakelam M. J., Woscholski R., 2012 Acute manipulation of diacylglycerol reveals roles in nuclear envelope assembly & endoplasmic reticulum morphology. PLoS One. 7: e51150. 23227247 10.1371/journal.pone.0051150 PMC3515572

[19] Fili N., Calleja V., Woscholski R., Parker P. J., and Larijani B. 2006 Compartmental signal modulation: Endosomal phosphatidylinositol 3-phosphate controls endosome morphology and selective cargo sorting. Proc. Natl. Acad. Sci. USA. 103: 15473–15478. 17030795 10.1073/pnas.0607040103 PMC1622847

[20] Hammond G. R., Fischer M. J., Anderson K. E., Holdich J., Koteci A., Balla T., and Irvine R. F. 2012 PI4P and PI(4,5)P2 are essential but independent lipid determinants of membrane identity. Science. 337: 727–730. 22722250 10.1126/science.1222483 PMC3646512

[21] Deerinck T. J., Bushong E. A., Thor A., and Ellisman M. H. 2010 NCMIR methods for 3D EM: a new protocol for preparation of biological specimens for serial block face scanning electron microscopy. Microscopy. Available from: www.ncmir.ucsd.edu/sbem-protocol.

[22] Kremer J. R., Mastronarde D. N., and McIntosh J. R. 1996 Computer visualization of three-dimensional image data using IMOD. J. Struct. Biol. 116: 71–76. 8742726 10.1006/jsbi.1996.0013

[23] Schindelin J., Arganda-Carreras I., Frise E., Kaynig V., Longair M., Pietzsch T., Preibisch S., Rueden C., Saalfeld S., Schmid B., 2012 Fiji: an open-source platform for biological-image analysis. Nat. Methods. 9: 676–682. 22743772 10.1038/nmeth.2019 PMC3855844

[24] Knorr R. L., Dimova R., and Lipowsky R. 2012 Curvature of double-membrane organelles generated by changes in membrane size and composition. PLoS One. 7: e32753. 22427874 10.1371/journal.pone.0032753 PMC3299685

[25] Shu X., Lev-Ram V., Deerinck T. J., Qi Y., Ramko E. B., Davidson M. W., Jin Y., Ellisman M. H., and Tsien R. Y. 2011 A genetically encoded tag for correlated light and electron microscopy of intact cells, tissues, and organisms. PLoS Biol. 9: e1001041. 21483721 10.1371/journal.pbio.1001041 PMC3071375

[26] Ulitzur N., and Gruenbaum Y. 1989 Nuclear envelope assembly around sperm chromatin in cell-free preparations from Drosophila embryos. FEBS Lett. 259: 113–116. 2557241 10.1016/0014-5793(89)81507-8

[27] Zhang B., and Zhai Z. H. 1995 The roles of two kinds of membrane vesicles in the formation of annulate lamellae and nuclear envelopes in a cell-free system from Xenopus egg extracts. [Article in Chinese] Shi Yan Sheng Wu Xue Bao. 28: 41–53. 7597869

[28] Cameron L. A., and Poccia D. L. 1994 In vitro development of the sea urchin male pronucleus. Dev. Biol. 162: 568–578. 8150215 10.1006/dbio.1994.1110

[29] Collas P., and Poccia D. 1996 Distinct egg membrane vesicles differing in binding and fusion properties contribute to sea urchin male pronuclear envelopes formed in vitro. J. Cell Sci. 109: 1275–1283. 8799817 10.1242/jcs.109.6.1275

[30] Peddie C. J., Blight K., Wilson E., Melia C., Marrison J., Carzaniga R., Domart M. C., O’Toole P., Larijani B., and Collinson L. M. 2014 Correlative and integrated light and electron microscopy of in-resin GFP fluorescence, used to localise diacylglycerol in mammalian cells. Ultramicroscopy. 143: 3–14. 24637200 10.1016/j.ultramic.2014.02.001 PMC4045205

[31] De Matteis M., Godi A., and Corda D. 2002 Phosphoinositides and the Golgi complex. Curr. Opin. Cell Biol. 14: 434–447. 12383794 10.1016/s0955-0674(02)00357-5

[32] Sicart A., Katan M., Egea G., and Sarri E. 2015 PLCγ1 participates in protein transport and diacylglycerol production triggered by cargo arrival at the Golgi. Traffic. 16: 250–266. 25491205 10.1111/tra.12246

[33] Persico A., Cervigni R. I., Barretta M. L., and Colanzi A. 2009 Mitotic inheritance of the Golgi complex. FEBS Lett. 583: 3857–3862. 19879264 10.1016/j.febslet.2009.10.077

[34] Altan-Bonnet N., Sougrat R., Liu W., Snapp E. L., Ward T., and Lippincott-Schwartz J. 2006 Golgi inheritance in mammalian cells is mediated through endoplasmic reticulum export activities. Mol. Biol. Cell. 17: 990–1005. 16314396 10.1091/mbc.E05-02-0155 PMC1356606

[35] Poccia D., and Collas P. 1997 Nuclear envelope dynamics during male pronuclear development. Dev. Growth Differ. 39: 541–550. 9338589 10.1046/j.1440-169x.1997.t01-4-00001.x

[36] Yang L., Guan T., and Gerace L. 1997 Integral membrane proteins of the nuclear envelope are dispersed throughout the endoplasmic reticulum during mitosis. J. Cell Biol. 137: 1199–1210. 9182656 10.1083/jcb.137.6.1199 PMC2132536

[37] Chia P. Z., and Gleeson P. A. 2014 Membrane tethering. F1000Prime Rep. 6: 74. 25343031 10.12703/P6-74 PMC4166942

[38] Sztul E., and Lupashin V. 2006 Role of tethering factors in secretory membrane traffic. Am. J. Physiol. Cell Physiol. 290: C11–C26. 16338975 10.1152/ajpcell.00293.2005

[39] Barroso M., Nelson D. S., and Sztul E. 1995 Transcytosis-associated protein (TAP)/p115 is a general fusion factor required for binding of vesicles to acceptor membranes. Proc. Natl. Acad. Sci. USA. 92: 527–531. 7831324 10.1073/pnas.92.2.527 PMC42774

[40] Grabski R., Hay J., and Sztul E. 2012 Tethering factor P115: a new model for tether-SNARE interactions. Bioarchitecture. 2: 175–180. 22992751 10.4161/bioa.21702 PMC3696062

[41] Levine T. P., Rabouille C., Kieckbusch R. H., and Warren G. 1996 Binding of the vesicle docking protein p115 to Golgi membranes is inhibited under mitotic conditions. J. Biol. Chem. 271: 17304–17311. 8663393 10.1074/jbc.271.29.17304

[42] Kasmapour B., Gronow A., Bleck C. K., Hong W., and Gutierrez M. G. 2012 Size-dependent mechanism of cargo sorting during lysosome-phagosome fusion is controlled by Rab34. Proc. Natl. Acad. Sci. USA. 109: 20485–20490. 23197834 10.1073/pnas.1206811109 PMC3528610

[43] Basu J., Betz A., Brose N., and Rosenmund C. 2007 Munc13-1 C1 domain activation lowers the energy barrier for synaptic vesicle fusion. J. Neurosci. 27: 1200–1210. 17267576 10.1523/JNEUROSCI.4908-06.2007 PMC6673179

[44] Ren Q., Wimmer C., Chicka M. C., Ye S., Ren Y., Hughson F. M., and Whiteheart S. W. 2010 Munc13-4 is a limiting factor in the pathway required for platelet granule release and hemostasis. Blood. 116: 869–877. 20435885 10.1182/blood-2010-02-270934 PMC2924225

