# Supplementary material for: Acute depletion of diacylglycerol from the cis-Golgi affects localized nuclear envelope morphology during mitosis
Source: J Lipid Res. 2018 Jun 12;59(8):1402–13. doi: 10.1194/jlr.M083899 (PMC6071775; doi:10.1194/jlr.M083899)
Supplement: Supplemental Data [file supp_59_8_1402__index.html]

Acute Depletion of Diacylglycerol from the Cis-Golgi Affects Localised Nuclear Envelope Morphology During Mitosis — Acute depletion of diacylglycerol from the cis-Golgi affects localized nuclear envelope morphology during mitosis — Supplemental Data 

# Acute depletion of diacylglycerol from the *cis*-Golgi affects localized nuclear envelope morphology during mitosis

## Supplemental Data

- movie A (.mov, 448 KB) - movie A
- Movie B (.mov, 2.7 MB) - Movie B
