# Supplementary material for: Acute depletion of diacylglycerol from the cis-Golgi affects localized nuclear envelope morphology during mitosis
Source: J Lipid Res. 2018 Jun 12;59(8):1402–13. doi: 10.1194/jlr.M083899 (PMC6071775; doi:10.1194/jlr.M083899)
Supplement: Supplemental Data [file supp_59_8_1402_v2_index.html]

Acute depletion of diacylglycerol from the cis-Golgi affects localized nuclear envelope morphology during mitosis — Supplemental Data 

# Acute depletion of diacylglycerol from the *cis*-Golgi affects localized nuclear envelope morphology during mitosis

## Supplemental Data

- Supplemental Figures 1 to 3 (.pdf, 9.7 MB) - Supplemental Figure S1 Supplemental Figure S2 Supplemental Figure S3
