# Supplementary material for: Acute depletion of diacylglycerol from the cis-Golgi affects localized nuclear envelope morphology during mitosis
Source: J Lipid Res. 2018 Jun 12;59(8):1402–13. doi: 10.1194/jlr.M083899 (PMC6071775; doi:10.1194/jlr.M083899)
Supplement: Supplemental Data [file 10.1194_M083899_jlr.M083899-1.pdf.html]

404 Not Found

# Not Found

The requested URL /jlr/suppl/2018/06/12/jlr.M083899.DC1/jlr.M083899-1.pdf was not found on this server.
